# Remote sensing with machine learning for multi-decadal surface water monitoring in Ethiopia

**DOI:** 10.1038/s41598-025-96955-y

**Published:** 2025-04-11

**Authors:** Mathias Tesfaye, Lutz Breuer

**Affiliations:** 1 Institute for Landscape Ecology and Resources Management (ILR), Research Centre for BioSystems, Land Use and Nutrition (iFZ), Justus Liebig University Giessen, Giessen, Germany; 2 Centre for International Development and Environmental Research (ZEU), Justus Liebig University Giessen, Giessen, Germany

**Keywords:** Surface water dynamics, SDG 6, Remote sensing, Google Earth Engine, Machine learning, Ecology, Environmental sciences, Hydrology

## Abstract

Monitoring the temporal evolution of surface water distribution is crucial to support surface water management and conservation, and could also effectively contribute to the achievement of Sustainable Development Goal 6 (SDG 6) ‘Clean Water and Sanitation’ at the regional level. Despite its importance, there is a lack of an operational method for determining surface water extent that also shows the interannual variability in Ethiopia. We assess Gradient Tree Boosting (GTB), Support Vector Machines (SVM), and Random Forest (RF) running on the Google Earth Engine (GEE) using Landsat for surface water monitoring at four sites in Ethiopia from 1986 to 2023. The results show that GTB, RF, and SVM have excellent classification accuracies, with overall, producer, and user accuracies consistently above 90%. GTB slightly outperforms the other two machine learning methods. The estimated water cover for our study sites shows a high degree of agreement with a benchmark dataset from the Joint Research Center (JRC), as indicated by coefficient of determination (R^2^) > 0.9 and root mean square percentage error (RMSPE) < 1%. The surface water dynamics of the four study sites depict a long-term increasing trend from 1986 to 2023, characterized by notable inter-annual variability. We identify the locations of this variability by analyzing the frequency of water occurrence over time and find that 84–94% are permanent water bodies, with the remaining water surface area changing over time. Mann–Kendall trend analysis does not confirm a general pattern over time for the four sites, suggesting that local site characteristics, water management and anthropogenic impacts are superimposed on the likely effects of climate change. Therefore, our results provide spatiotemporal information for surface water monitoring to support water resource management and policy in Ethiopia. This could also effectively contribute to the sustainable use and achievement of SDG 6 at the regional level.

## Introduction

Surface water is an important natural resource that is subject to considerable spatial and temporal dynamics. It comprises a range of inland water bodies, including rivers, streams, ponds, reservoirs, lakes and wetlands^1,2^. They are a critical components of water resources for humans and terrestrial ecosystems, covering approximately 3% of the world’s land area^3^. In addition to maintaining biodiversity and providing essential and diverse ecosystem services^4–6^, surface waters play a crucial role in the climate system and the water cycle^7,8^. However, the biodiversity of these ecosystems is rapidly declining^9^, exacerbated by climate-induced hydrological extremes such as floods or droughts and the emergence of water-related diseases, both of which contribute to escalating loss of life. Therefore, timely monitoring of surface water dynamics is crucial for informed policy and decision-making processes to ensure its sustainable use and management^10,11^.

**Fig. 1:**
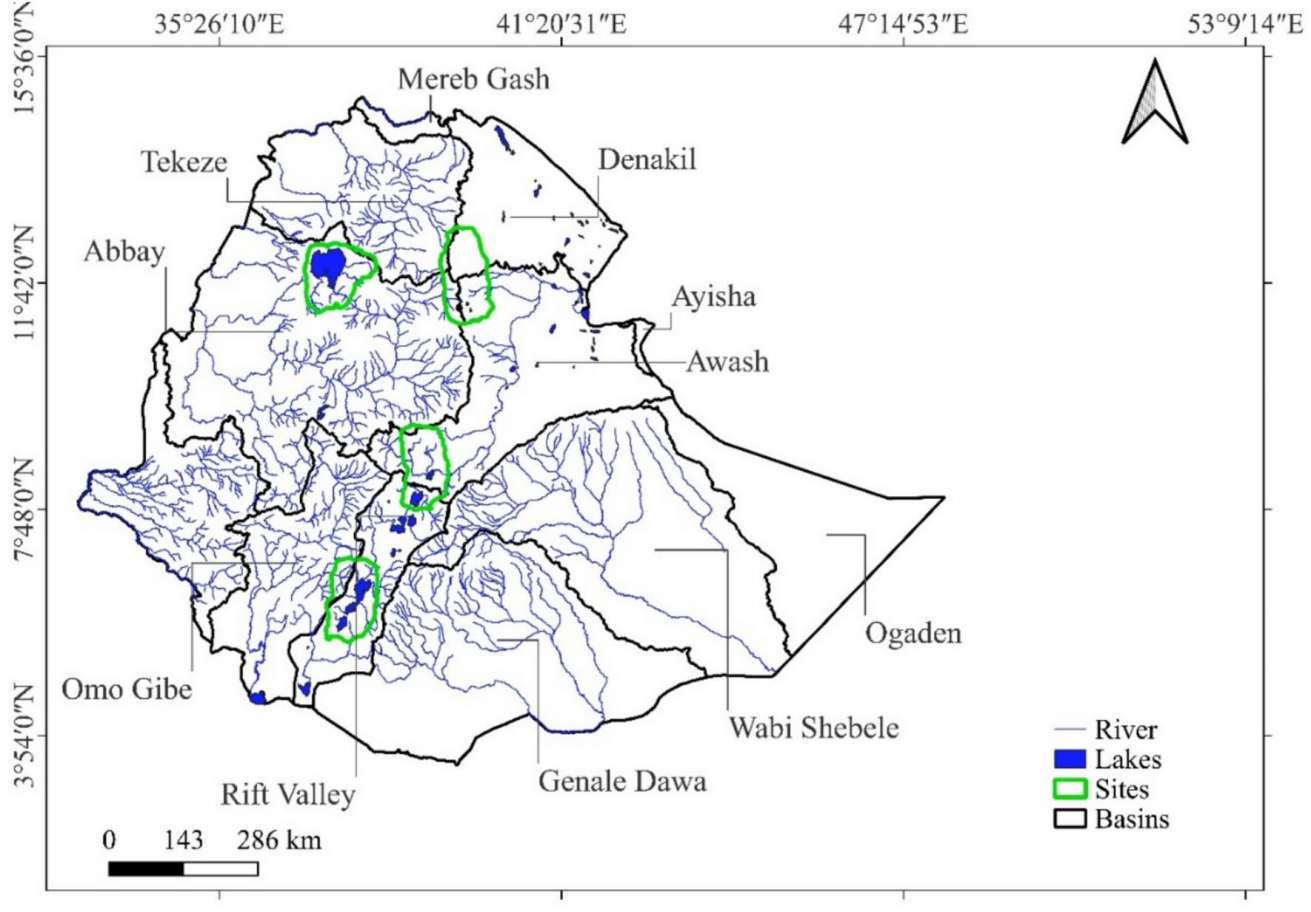
Location of the study areas with the main water bodies of Ethiopia.

Ethiopia has substantial surface water resources, comprising eight river basins, one lake basin, and three arid basins (Fig. 1). A notable rainfall gradient separates the central and western highlands, which receive abundant annual rainfall of up to 1200 mm, from the arid southeastern, eastern, and northeastern regions, which receive 200 mm and less^12^. As a result, surface water resources are less abundant in the eastern part of Ethiopia, particularly in the Awash Basin, while the western part, including the Abay (Blue Nile) River Basin, has substantial water resources. Despite the critical importance of water resources to both human well-being and the country’s ecosystems, monitoring systems for surface water availability and hydrological flows are in a poor state and have sometimes proven to be unreliable or dysfunctional^13^. A comprehensive assessment of a long-term indicator of surface water resources, as required by SDG 6, is currently lacking.

Although Ethiopia is recognized as having abundant water resources, this potential is not fully exploited and translated into basin planning and development due to technical constraints and inefficient management in the water sector^12^. Flooding is particularly prevalent in the lowlands of parts of the Awash river basin in Ethiopia^14^ and has resulted in crop damage, loss of soil nutrients and erosion^15^. Water-related ecosystems in Ethiopia are experiencing various challenges including inter-annual climate variability and changing water regimes ^16^ or increasing sedimentation^17,18^. Thus, timely monitoring of surface water resources and detection of spatio-temporal changes is critical for sustainable use and management of the country’s water resources.

Satellite remote sensing provides the ability to continuously monitor surface water at multiple spatio-temporal scales^1,19,20^. This can fill in the gaps in poorly mapped areas. Landsat imagery is the primary data source for long-term surface water monitoring due to its consistency, free access, and long-time availability^1,21^.

Recent advances in remote sensing technology, characterized by high spatial, temporal and spectral resolution, have contributed substantially to the monitoring of surface waters, with a focus on their accurate detection^22^. However, monitoring lakes and reservoirs and providing accurate spatio-temporal information on the dynamics of surface water extent is still a challenge for classification algorithms due to their characteristics. This challenge is demonstrated for example for Lake Mead (USA), Nova Ponte reservoirs (Brazil) and Lake Williston (Canada) due to their temporal variability, different narrow ends, dynamic shape, and missing values due to cloud or ice cover^23^. Therefore, this study explores the potential of machine learning to detect and monitor surface water bodies using Landsat data in a cloud computing platform.

Large-scale surface water monitoring is a challenge in processing large amounts of remotely sensed imagery^24^. In recent years, Google Earth Engine (GEE) has become a cloud computing platform for analyzing large amounts of data, including global datasets. It provides access to a range of satellite imagery, particularly Landsat and Sentinel products, and facilitates high-performance computing for social and environmental analysis, including water monitoring^25^. Thus, in our study, we use GEE to process and analyze surface water information from Landsat imagery using machine learning.

Water indices based on a multi-band classification method are often used in remote sensing to detect surface water^26^. Water indices are quick and easy to calculate^27^. However, the spectral signatures of water bodies vary in space and time, making it challenging to select a common threshold for distinguishing water bodies from non-water bodies^20^. These problems can be overcome by using machine learning algorithms such as Gradient Tree Boost (GTB), Random Forest (RF) and Support Vector Machine (SVM).

Several studies have explored the detection and dynamics of surface water, leveraging advanced machine learning and remote sensing techniques. For instance, a random forest approach was applied to almost four decades of satellite data to analyze surface water variability in a semi-arid region of Australia^20^. In the same way, spatio-temporal variations in surface water from 1985 to 2020 have been investigated using machine learning algorithms applied to time-series remote sensing datasets^28^. In the United States, a gradient-boosted regression model has been employed to distinguish between open and vegetated water bodies, providing enhanced classification accuracy^29^. Australia has also been the focus of large-scale, multi-year surface water mapping efforts, offering high-resolution spatio-temporal data critical for regional planning and water resource management^30^. Moreover, the hydrological dynamics of Ethiopia’s major lakes have been assessed using RF and modified normalized water index, further contributing to our understanding of surface water fluctuations in diverse environments^31^. However, surface water monitoring and trends, including lakes surrounding rivers, ponds, reservoirs, and wetlands, has not been addressed. Despite the advancements, existing studies primarily focus on individual models applied to specific geographic regions. To the best of our knowledge, a comprehensive assessment of multiple modeling approaches across diverse geographical locations for surface water detection and long-term monitoring remains unexplored.

The JRC dataset provides surface water data for the entire globe^1^. However, for a regional application like Ethiopia, there are some limitations in the JRC data, such as data gaps and lack of detailed detection and analysis of local spatio-temporal variability in the operational application using machine learning with GEE. Nevertheless, the JRC data set allows to benchmarking new methods in surface water detection, which can then be used for gap filling or further regional analysis. Therefore, this study aims to (i) evaluate a number of machine learning methods (i.e., GTB, SVM, RF), (ii) compare the results with the JRC dataset for time series surface water monitoring, and (iii) statistically analyze inter-annual and long-term trends of surface water coverage in Ethiopia.

## Materials and methods

### Description of study area

Ethiopia has significant surface water resources and 12 major river basins (Fig. 1). Four of Ethiopia’s river basins, namely Abay or Blue Nile, Baro-Akobo, Tekeze, and Mereb, which are part of the Nile Basin, cover 33% of the country and drain the northern, central, and western parts^32^. The four river basins of Abay (Blue Nile), Tekeze, Baro Akobo, and Omo Gibe contain about 90% of Ethiopia’s water sources^33^. In the eastern part of Ethiopia, surface water resources are limited as there are almost no perennial rivers found below 1,500 m a.s.l. Three of the major basins (Aysha, Dinakle, and Ogaden) are mainly dry and have no permanent runoff^12^. Ethiopia’s 12 major lakes cover an area of about 7300 km^2^. Lake Tana (in the Abay Basin) is the largest and is the main source of water for the Abay River. Most of the other lakes are saline and located in the Rift Valley. In this study, four sites are selected due to the diverse availability of water resources including lakes, rivers, reservoirs and wetlands and their complex interactions with human activities across different geographical locations and basins of Ethiopia (Fig. 1). The first site is the Addis-Ziway site which includes Addis Ababa and its surroundings, Bishoftu lakes, Ziway and Koka reservoirs. The Hayq-Hashenge site is the second site covering the northeastern part of Ethiopia including Hayk, Hardibo and Hashenge. Thirdly, the Tana site covers mainly Lake Tana. Finally, the Abaya-Chamo site includes Arba-Minch and the lakes of Abaya and Chamo.

### Data acquisition and preprocessing

We utilize Tier 1 Landsat time series imagery retrieved from the United States Geological Survey (USGS) data provider, obtained through GEE’s data access from 1986 to 2023. We select Landsat imagery for its continuous access at 30 m spatial resolution over time and a 16-day revisit time. However, the Landsat sensor does not detect smaller water bodies due to its spatial resolution^1,34^ and might miss observations resulting in an underestimation of surface water extent. In this study, Landsat imagery includes the Thematic Mapper (TM) sensor for Landsat 5 covers from 1986 to 1998; the Enhanced Thematic Mapper Plus (ETM +) for Landsat 7 between 1999 and 2012 and the Operational Land Imager (OLI) and Thermal Infrared Sensor (TIRS) for Landsat 8 and OLI-2 and TIRS-2 for Landsat 9 covers from 2013 to 2021 and 2022 to 2023, respectively. For each time step, the feature vector consists of multiple spectral bands, including quality assurance (QA) bands to providing important information for surface water detection and improving classification accuracy. The spectral bands included in the dataset cover various portions of the electromagnetic spectrum, supporting accurate distinguishing of water bodies from other land cover types. The blue band covers wavelength ranges from 0.45 to 0.52 µm in Band 1 for Landsat 5/7 and 0.45–0.51 µm in Band 2 for Landsat 8/9. The green band captures wavelengths from 0.52–0.60 µm in Band 2 for Landsat 5/7 and 0.53–0.59 µm in Band 3 for Landsat 8/9. The red band represents Band 3 (0.63–0.69 µm) for Landsat 5/7 and Band 4 (0.64–0.67 μm) for Landsat 8/9. The near-infrared (NIR) band covers wavelengths from 0.77 to 0.90 µm in Band 5 for Landsat 5/7 and 0.85–0.88 μm in Band 5 for Landsat 8/9. The shortwave infrared 1 (SWIR1) band represents Band 6 (1.55–1.75 µm) for Landsat 5/7 and Band 6 (1.57–1.65 μm) for Landsat 8/9. The Short Wave Infrared 2 (SWIR2) band covers wavelengths from 2.09 to 2.35 µm in band 7 for Landsat 5/7 and 2.11–2.29 μm in Band 7 for Landsat 8/9. In addition to spectral bands, QA bands are included into the feature vector to enable cloud and shadow masking, thereby enhancing classification accuracy.

The standard Landsat Ecosystem Disturbance Adaptive Processing System (LEDAPS) algorithm is used to produce surface reflectance data for Landsat 5 and Landsat 7^35,36^. The Landsat surface reflectance code (LaSRC) algorithm is used to produce surface reflectance data for Landsat 8^37^. Landsat 9 surface reflectance is also produced using the LaSRC algorithm. These products are radiometrically and geometrically corrected. We use the GEE cloud computing platform to preprocess Landsat time series imagery. We chose GEE for its ability to handle large remotely sensed data and long-term time series analysis^25^. The USGS L7 Phase-2 Gap Filling Protocol^38^ is employed to correct scan line gaps for Landsat 7 imagery acquired after 2003. We combine the multiple Landsat images into a single annual composite per site using the GEE median reducer function^39^. The data are preprocessed to include blue, green, red, near-infrared, shortwave infrared 1, and shortwave infrared 2 spectral bands, which are primarily used for water detection^40^, as well as quality assurance (QA) bands for cloud and shadow masking. The CFmask algorithm^41^ is used for cloud and shadow masking to ensure data quality. This process identifies pixels affected by clouds or shadows and excludes them from further analysis using the QA_PIXEL band.

### Machine learning for surface water detection

Machine learning algorithms are employed to detect, and quantify the extent of surface water, like to other studies^20,29,42,43^. Training samples are used to train the machine learning for surface water detection and area estimation using time series Landsat imagery, and validation samples are used to assess their accuracy. These sample points are collected at four sites using a stratified random sampling approach from water and non-water areas, respectively. Stratified random sampling approach is mainly used in remote sensing based classification because smaller classes can be adequately represented regardless of the size of the features and their spatial extent^44^. Spatial stratification also improves the classification accuracy of remote sensing^45^. For example, stratified random sampling involves dividing the study area into distinct strata of water and non-water. The water and non-water training sample points are collected from the satellite images from 1986 to 2023. These samples are collected manually through visual interpretation similar to previous studies^20,29,31^ considering the type, size, color, and pattern of inland water bodies including rivers, streams, ponds, reservoirs, lakes, and wetlands^1,34^ and non-water bodies defined except these features. Then, random samples are drawn independently from each layer to ensure balanced and accurate representation of the areas and to train the model. Water pixels are collected from the sites Addis-Ziway, Hayq-Hashenge, Tana, and Abaya-Chamo, with a number of 1086, 1124, 1218, and 1036, respectively. The non-water pixels from the same sites are 1091, 1228, 1025, and 1038, respectively. Thus, a total of 4464 water pixels and 4382 non-water pixels are used to train each model.

We use the GEE Python application programming interface (API) and the geemap package to access, process, and analyze the Landsat images in the Google Colab environment. We select GTB, RF, and SVM as widely used and efficient tools for remotely sensing surface water detection across space and time. For example, GTB was used to detect changes in open water and vegetated water at 12 different sites from 2017 to 2021^29^. Similarly, RF has been used to assess surface water dynamics in semi-arid regions at sub-continental scale from 1986 to 2011, where it demonstrated its robustness and efficiency in handling remote sensing data^20^. In addition, SVM has been shown to be effective in quantitative and qualitative assessment of the detection of large inland water bodies^46^. Each machine learning method is briefly described below:

(1) GTB is an ensemble learning method that builds a predictive model in the form of an ensemble of weak learners into a stronger ensemble using gradient descent optimization to minimize the loss function^47^. This is done by successively refining a model by adding new trees to correct errors. This sequential nature distinguishes GTB from other ensemble methods such as RF, where trees are built independently. In GTB, each new tree is trained to predict the residuals (errors) of the previous combined ensemble. The ensemble is then updated by adding a weighted version of the new tree. This iterative process continues until a predefined number of trees is reached.

The estimation function *f(x)* maps x to *y* while considering for the joint distribution of all (*y,x*) values to model the relationship between a random output (or response) variable *y*, and a set of random input (or explanatory) variables *x* = *{*× *1,…, xn}*^47^. Given a training dataset.

$\left\{ {{\text{yi}},{\text{xi}}} \right\}_{i = 1}^{N}$ with known values of *y* and *x*, *X* = {× 1, × 2,…, *xn*} is the features of Landsat data (spectral bands) while *Y* = {*y*1,*y*2,…, *yn*} is the label (water = 1, no-water = 0). The algorithm starts by initializing the model *F*_*0*_*(x)* that minimizes the loss function $\psi$ (*y*_*i*_*,γ*) over all training samples (*xi,yi*) in Eq. (1).

1 $$F_{0} \left( x \right) \, = \, \arg \, \min \gamma \sum\nolimits_{i - 1}^{N} {\psi \left( {yi,\gamma } \right)}$$

The algorithm builds *M* trees sequentially, where each tree corrects the errors of the previous ones. Thus, it iterates for *m* = 1 to *M* (number of trees). For each training sample *i*, the pseudo-residual *Ỹim* is calculated, which is the negative gradient of the loss function $\psi$ regarding to the current model *Fm* − 1(*x*) Eq. (2).

2 $$\tilde{Y}im = - \left[ {\frac{{\partial \psi \left( {yi,F\left( {xi} \right)} \right)}}{{\partial F\left( {xi} \right)}}} \right]F\left( x \right) = Fm - 1,i = 1,N$$

In the next step, a decision tree with *L* terminal nodes (leaves) is fit to the pseudo-residuals Ỹ*im* using the input features *xi* Eq. (3).

3 $$\left\{ {Rlm} \right\}_{1}^{L} = L - ter\min al node tree\left\{ {\tilde{Y}im,xi} \right\}_{1}^{N}$$

For each leaf region *Rlm*, the optimal value *γlm* that minimizes the loss function is calculated when added to the current model *Fm* − 1(*x*) Eq. (4).

4 $$\gamma lm \, = \, \arg \, \min \mathop \sum \limits_{xi \in Rlm} \psi (yi,Fm - 1\left( {xi + \gamma } \right)$$

Following, the model is updated by adding the predictions of the new tree, weighted by a learning rate *ν* (shrinkage parameter) Eq. (5).

5 $$F_{m} \left( x \right) \, = \, F_{m - 1} \left( x \right) + \nu \cdot \gamma lm1\left( {x \in Rlm} \right)$$

The number of decision trees depends on the complexity of the model. A larger number of decision trees can result in a more accurate but more computationally intensive model. The shrinkage parameter controls the contribution of each tree to the model and helps prevent overfitting, while the maximum node parameter limits the size of individual trees to avoid overly complex structures. This combination of parameters aims to strike a balance between model accuracy and computational efficiency. This makes it suitable for surface water detection in GEE. We conduct hyperparameter tuning for the GTB classifier using a grid search with tenfold cross-validation, evaluating combinations of the decision trees (100, 300, 500), learning rate (0.01, 0.05 and 0.2), maximum leaf nodes (5, 10 and 20), sampling rate (0.6, 0.7 and 1.0), and loss (least square and least absolute deviation). The algorithms arrive at the final hyperparameter values of 500 for decision trees, 0.05 for learning rate, 20 for maximum leaf nodes, 1.0 for sampling rate and least square loss function.

(2) RF is a non-parametric method that can deal with non-linear and large data sets. The RF builds the trees in parallel procedures that are fully grown, and each tree is used to predict the missing observations that do not occur in a bootstrap sample^48^. The average of all predictions outputs is used to calculate the predicted class of an out-of-bag observation^48^. We undertake hyperparameter tuning by grid search with tenfold cross-validation, evaluating combinations of the decision trees (100, 300, 500), minimum leaf population (1, 5 and 10), bag fraction (0.6, 0,8 and 1.0) and maximum leaf nodes (5, 10 and 20). The algorithms arrive at the optimal hyperparameter values of 100 for decision trees, 1 for minimum leaf population, 0.6 for bag fraction, 5 for maximum leaf nodes.

RF is an ensemble classification model which mainly uses trees structured classifiers {*h(x,Θk), k* = *1,…*}, where the {*Θk*} are independent and identically distributed random vectors and *x* is an input^48,49^. Each tree h(*x,Θk*) contributes a single vote for the most frequently occurring class at input x and the final classification is assigned based on the majority vote across all trees in Eq. (6)^49^. For classification, *Ĉb(x)* be the class prediction of the b-th tree in the random forest. Then, the final random forest classification is given by:

6 $$\overset{\lower0.5em\hbox{$\smash{\scriptscriptstyle\frown}$}}{C} b\left( x \right) = majority\,vote\left\{ {\overset{\lower0.5em\hbox{$\smash{\scriptscriptstyle\frown}$}}{C} b\left( x \right)} \right\}_{b = 1}^{B}$$

where *B* is the total number of trees in the forest.

(3) The SVM finds a hyperplane or set of hyperplanes in infinite dimensional space that uniquely classifies the data points and finds the optimal separating hyperplanes^50^. The balance between achieving a smooth decision boundary and correctly classifying training points is controlled by the regularization parameter cost. The parameter gamma is also relevant to the flexibility of the decision boundary. The choice of the kernel type is important which includes linear, polynomial and radial basis function. Therefore, we choose the polynomial kernel among linear and radial basis function based on the higher classification accuracy results in distinguishing water from non-water pixels. The degree parameter is specific to the polynomial kernel. The hyperparameters of the polynomial kernel allow fine-tuning of the decision boundary using a grid search with tenfold cross-validation, evaluating the combination of gamma values (0.0001, 0.001, 0.01, and 0.1), cost values (0.1, 1, 10, 100, 1000), and degree (2, 3, and 4). The algorithms arrive at the optimal values of 0.0001, 0.1, and 2 for gamma, cost, and degree, respectively, to ensure that the model achieves high accuracy.

The optimal hyperplane algorithm consists of the labeled training patterns *(Y1, X1), …..,(Yl, Xl), Yi ∈ {-1, 1}*, which is linearly separable when there is a vector *w* and a scalar *b* satisfying the inequalities for all items of the training dataset in Eqs. (7), (8)^51,52^.

7 $$w \cdot xi + \, b \, \ge \, 1 \, if yi \, = 1$$

8 $$w \cdot xi \, + \, b \, \le - 1 \, if yi = - 1$$

The above equation can be written, combining the inequalities as Eq. (9).

9 $$yi(w \cdot xi \, + b) \, \ge \, 1, \, i = 1, \ldots ,l$$

The standard optimization method is used and the Lagrangian is constructed in Eq. (10)

10 $$L\left( {w, \, b, \wedge } \right) \, = w \cdot w \, - \mathop \sum \limits_{i = 1}^{l} \alpha i\left[ {yi\left( {xi \cdot w + b} \right) - 1} \right],$$

where $\wedge T$ = *(*$\alpha 1, \ldots .,\alpha l){ }$ is the vector of Lagrange multipliers that incorporates the constraints. The decision rule separating the two categories is written as follows Eq. (11)

11 $$f\left( x \right) = sign\left( {\mathop \sum \limits_{\sup port vector} yi\alpha^{0} \left( {xi^{\prime } x} \right) - b^{0} } \right)$$

where *α*^*0*^ and *b*^*0*^ are the optimized Lagrange multipliers and bias term, respectively. As mentioned before, we use a polynomial kernel given in Eq. (12).

12 $$K\left( {x1,x2} \right) = \left( {x1 {\prime} x2 + 1} \right)^{p}$$

where *p* is the degree of the polynomial kernel. Thus, this extends the decision rule to use the kernel function for non-linear classification according to Eq. (13).

13 $$f\left( x \right) = sign\left( {\mathop \sum \limits_{\sup port vector} yi\alpha^{0} K\left( {xi^{\prime } x} \right) - b^{0} } \right)$$

### Accuracy assessments

Accuracy assessments are carried out to determine whether or not the results of surface water detection and area estimation are acceptable. Validation samples are used to assess the accuracy of the three machine learning methods and the JRC dataset at our specific sites. Confusion matrices, the common method for describing classification accuracy ^53^, are used to compare the reference data and the corresponding classification results on a category-by-category basis. User’s accuracy, also referred to as reliability, measures the likelihood that a sample classified on the map accurately represents that class in reality, thereby assessing how well the map reflects the actual conditions on the ground^54^. Another metric, referred to as producer’s accuracy, shows the likelihood that a reference pixel is accurately categorized and evaluates how effectively a particular area is able to be categorized^55^. Overall accuracy gives a general measure of how well the classifier performs across all categories. These metrics are applied in many remote sensing image classification^20,34,43^ and are convenient for the interpretation of the classification (Foody, 2002).

We calculate the user’s accuracy, producer’s accuracy, and overall accuracy to assess the results of the surface water detection and area estimation. The user’s accuracy (UA) is calculated by dividing the number of correctly classified pixels in each category by the total number of pixels classified in that category (the row total). This is known as the specificity or true negative rate, and is the complement of the commission error ^53^. The producer’s accuracy (PA) is obtained by dividing the number of correctly classified pixels in each category (on the main diagonal) by the number of training set pixels used for that category (the column sum). This is known as the sensitivity, or true positive rate, and the complement of the omission error. The overall accuracy (OA) is a measure of how well an algorithm performs compared to reference the training data. It is computed as the percentage of correctly classified pixels versus total pixels. tenfold cross-validation is used to evaluate the performance of the model^56^.

### Assessing surface water area estimates with the JRC dataset

GEE is utilized for data processing, analysis, and visualization, leveraging its cloud computing capabilities to retrieve the JRC dataset. The JRC dataset provides continuous surface water data over the globe, although there are data gaps from 1988 to 1993, 1996–1997 and 2021–2023 in our study area during our study period from 1986 to 2023. Hence, we identify and exclude such data gaps through visual inspection of each site to assess and compare our estimated surface water area with the Joint JRC Global Surface Water Data (GSWD). A linear regression model, along with its metrics of R^2^, root mean square error (RMSE), mean absolute error (MAE), and RMSPE, is used to assess the agreement between the JRC dataset and our surface water area estimates.

### Spatio-temporal surface water dynamics and trends

We construct a time series product of surface water extent using continuous Landsat imagery for 38 consecutive years from 1986 to 2023. We use the Mann–Kendall test to analyze the long-term monotonic (increasing or decreasing) trends in various hydroclimate time series^57^. This test is robust and less sensitive to outliers, which enhances its applicability in the analysis of noisy data^58^. We assess the statistical significance of trends using the p-value and evaluate the strength of the relationship between the time trend and changes in the surface water area using Kendall’s Tau coefficient^59,60^. We also use Sen’s slope estimator to estimate the extent of the change in surface water area over time^61^. The Pettit test is applied to identify points of change or abrupt shifts in a time series of hydroclimatic datasets^62^. The percentage change in surface water area is calculated by taking the difference between the post-change and pre-change areas, dividing that difference by the pre-change area, and then multiplying the result by 100 to express the change as a percentage. This provides a quantitative assessment of the relative changes in surface water area over time. We further calculate the water occurrence frequency (WOF) for each site by determining the percentage of time during the study period that each pixel is classified as water using Eq. (1)^1,63^. The WOF classes are used to distinguish permanent water bodies from those with seasonal or temporary surface water cover. In particular, areas with a WOF value of > 60% are classified as permanent water bodies, indicating that the presence of surface water is permanent^28^. Field observations and verification are undertaken to ensure the spatial distribution and pattern of surface water. Python programming in the Google Colab environment is used to implement all tests and calculate WOF as:

14 $$WOF \left( \% \right) = \left( {\frac{{\sum wi^{t} }}{T}} \right) \cdot 100$$

where $\sum wi^{t}$ denotes the total count of water presence (pixels with a value of 1) for a specific pixel *i*, across all time steps *t*, and *T* denotes the total number of time steps (years) considered in the calculation.

## Result

### Machine learning for surface water detection

**Fig. 2:**
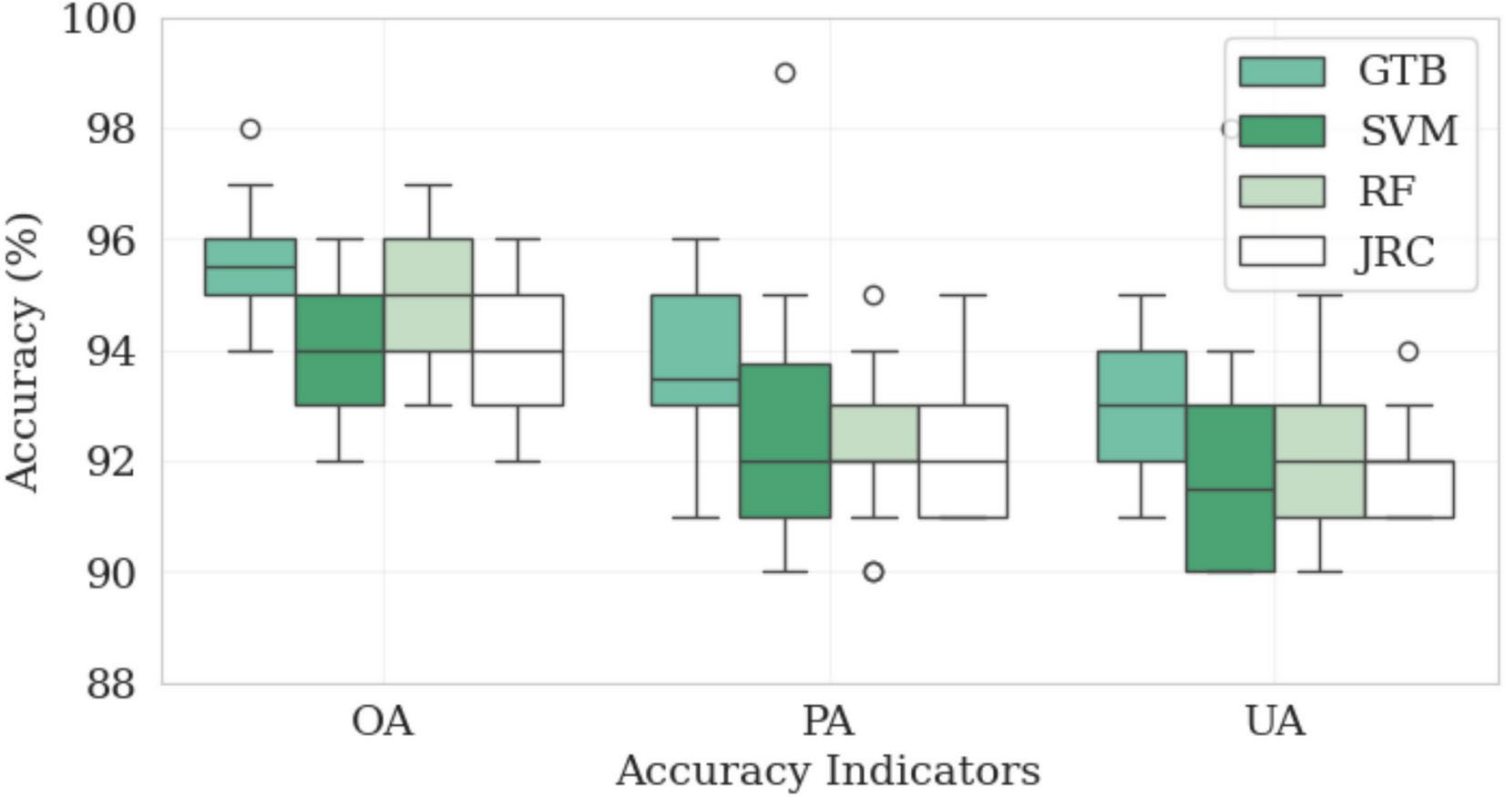
Accuracy assessment of surface water estimation using the machine learning methods random forest (RF), gradient tree boosting (GTB) and support vector machine (SVM) along with results obtained from the Joint Research Center (JRC) dataset in the Addis-Ziway site. *OA* overall accuracy, *PA* producer accuracy, *UA* user accuracy.

The remote sensing-based surface water detection of the Addis Ziway site using machine learning methods GTB, RF and SVM as well as results of the JRC data set depicts satisfactory classification accuracies of > 90% in terms of producer’s accuracy, user’s accuracy, and overall accuracy (Fig. 2). The GTB achieves slightly better accuracies with an overall accuracy of 94–98% from 1986 to 2023. However, RF and SVM also have high overall accuracies of 93% to 97% and 92% to 96%, respectively. Thus, these results highlight the effectiveness of all three machine learning models for surface water detection.

**Fig. 3:**
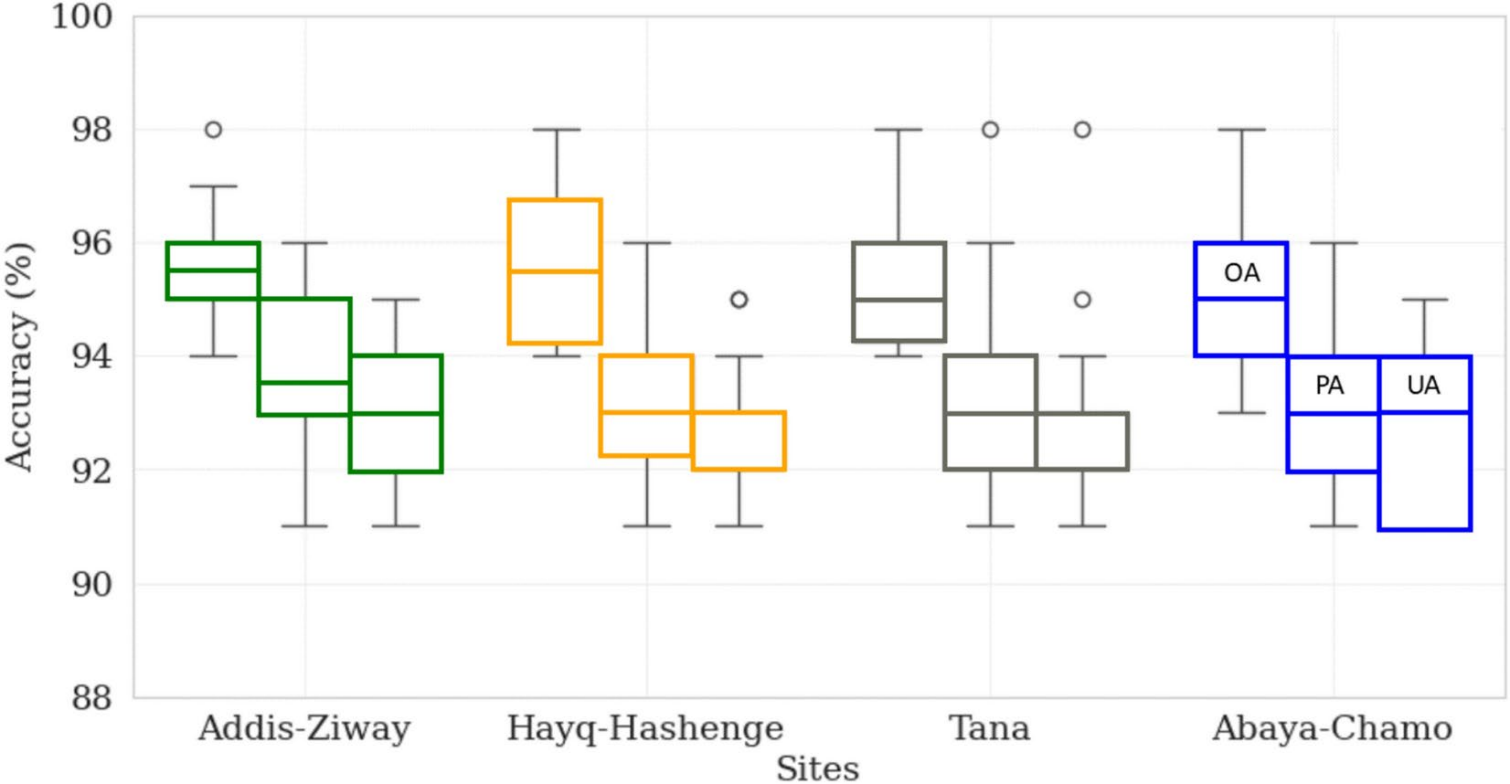
Accuracy assessment of the best performing machine learning method gradient tree boosting (GTB) at the four sites with overall accuracy (OA), producer accuracy (PA) and user accuracy (UA).

Due to its best performance, the GTB model is selected for further estimation of the surface water area for all four sites (Fig. 3) and subsequent analysis. At all of the sites, the three accuracy indicators range between 91 and 98% and thus at a very high and comparable level. The highest overall agreement between training data and the machine learning methods is found for Addis-Ziway and the lowest values for Abaya-Chamo. We attribute this to the high image quality and low cloudiness at the Addis-Ziway site. In addition, the separation of water from non-water bodies at the Abaya-Chamo site was more difficult, as water bodies often overlap with vegetation areas at this site. In general, the overall accuracies of the GTB method for all locations and all time periods is well over 90%.

### Assessing surface water area estimates with the JRC dataset

**Fig. 4:**
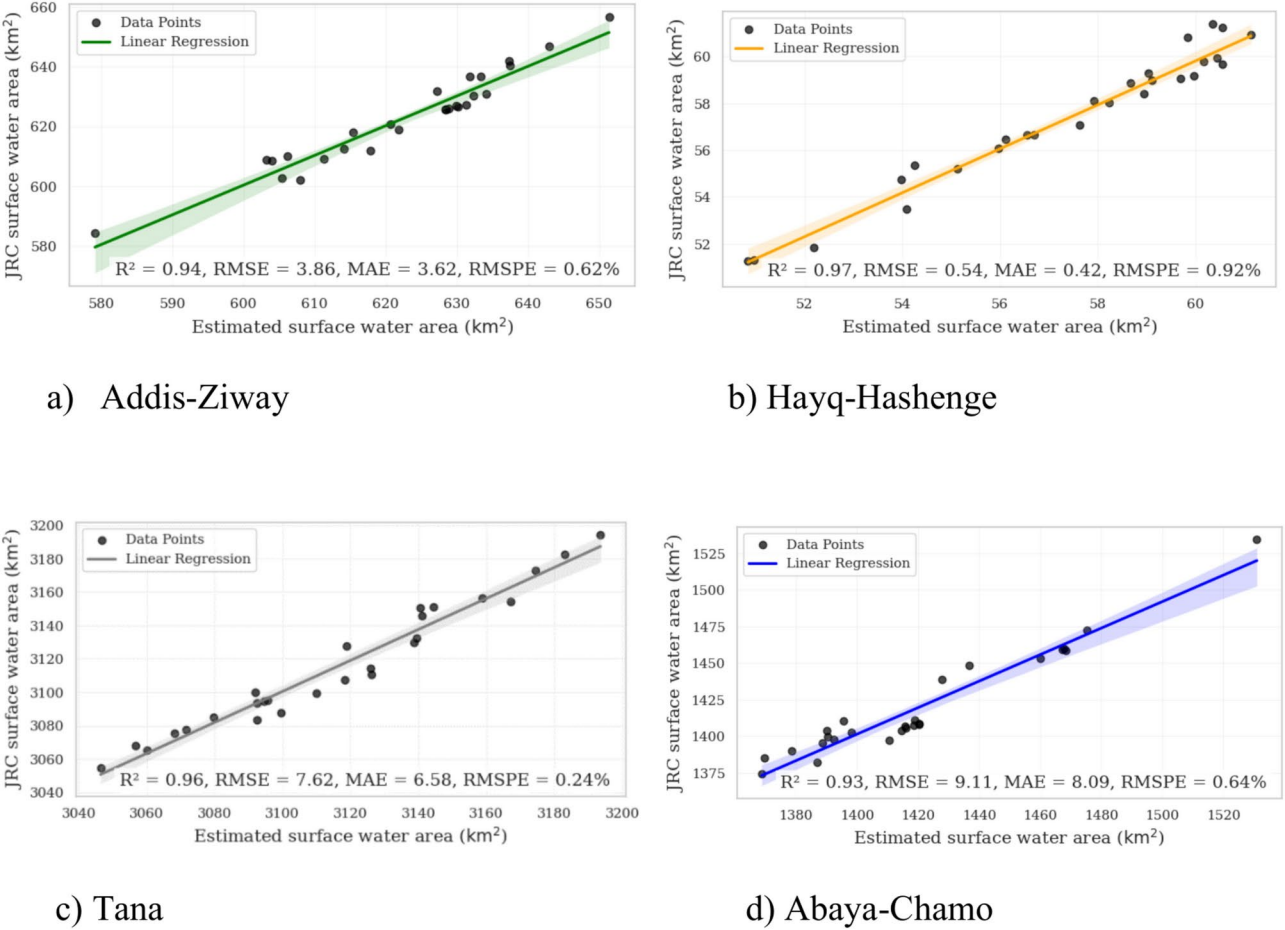
Correlation analyses and linear regression for comparison of the gradient tree boosting (GTB) estimated surface water area and the JRC dataset for the four sites.

The comparison between the estimated surface water area and the JRC dataset reveals a good agreement and the reliability of the machine learning models. This is evident by R^2^ values above 0.9 and RMSPE values below 1% for all sites (Fig. 4). The RMSE and MAE values range from 0.54 to 9.11 and 0.42 to 8.09, respectively. Hence, the absolute deviations between the machine learning estimated and JRC-derived surface water areas are low.

**Fig. 5:**
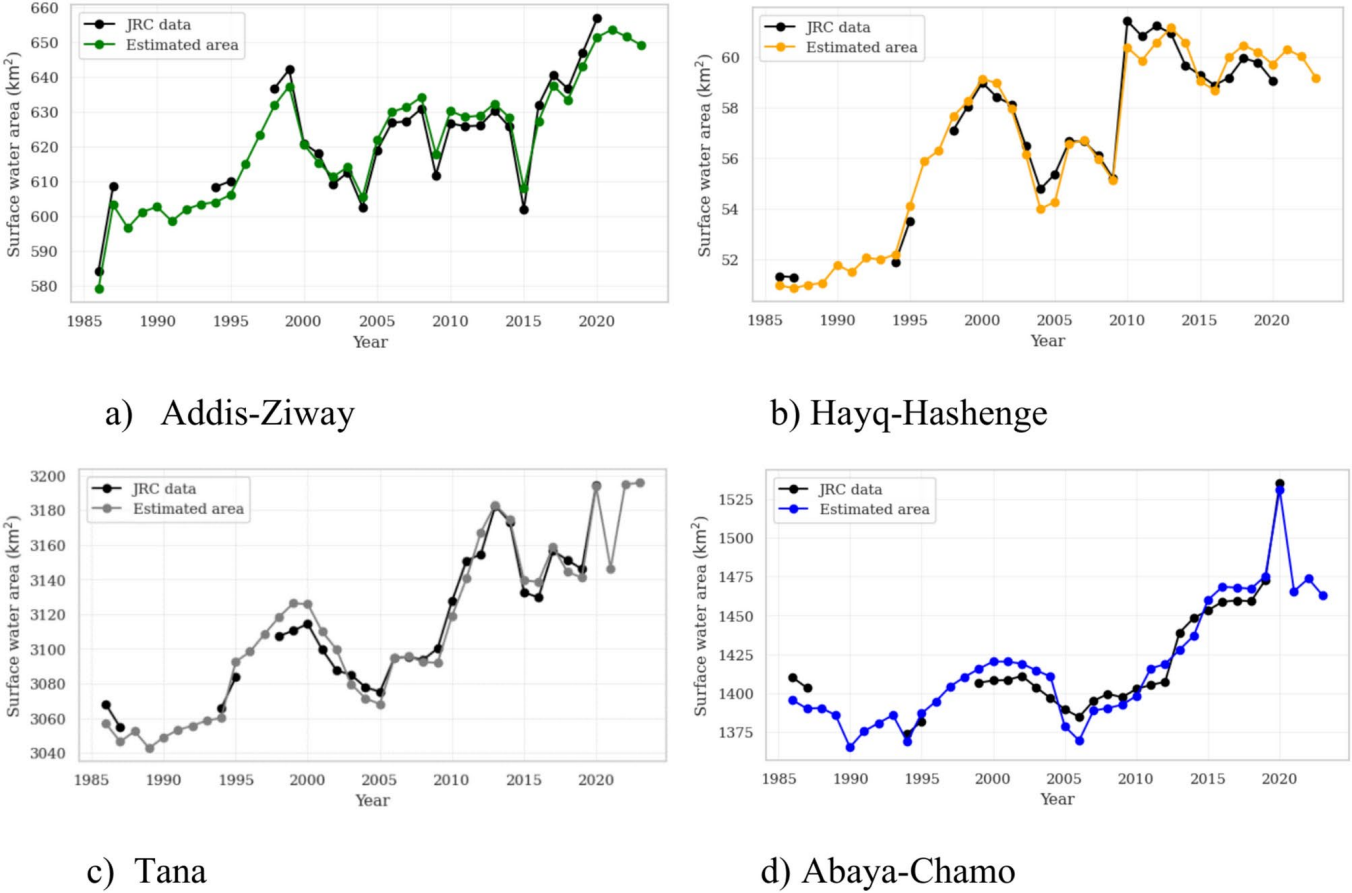
Comparison of time series of surface water area estimation based on the gradient tree boosting (GTB) method and the JRC dataset for all four sites. Note the data gaps of surface water extend in the JRC dataset from 1988–1993, 1996–1997 and 2021–2023.

In addition to the statistical agreement, Fig. 5 displays the time series for the estimated surface water area. As can be seen, the temporal development is well captured by the GTB algorithm. We do not see a general difference in the performance of GTB in small (Hayq-Hashenge), medium (Addis-Ziway) or large scale (Abaya-Chamo, Tana) test sites. But what we observe is a significant increase in surface water extent over the almost four decades for all locations, despite some major fluctuations between the years. For all four areas, we find an interim decrease until 2005 after an expansion of the water areas until the beginning of 2000. From 2005 on, the expansion of the water surface generally increases again. It is noticeable that there are subsequently increasing differences in the dynamics of the four areas, which in turn can be attributed to more regional trends in precipitation and evaporation. For example, in Abaya-Chamo there is no decrease or stagnation of the water surface area in 2009 as is the case elsewhere. The peak in 2020 in the same site is also striking. In Hayq-Hashenge, the dramatic increase of 9.51% from 2009 to 2010 is also more pronounced than in any other region. A Mann–Kendall test for monotony of trends is carried out to analyse the temporal trends further and to check for significant changes.

### Spatio-temporal surface water dynamics and trends

**Fig. 6:**
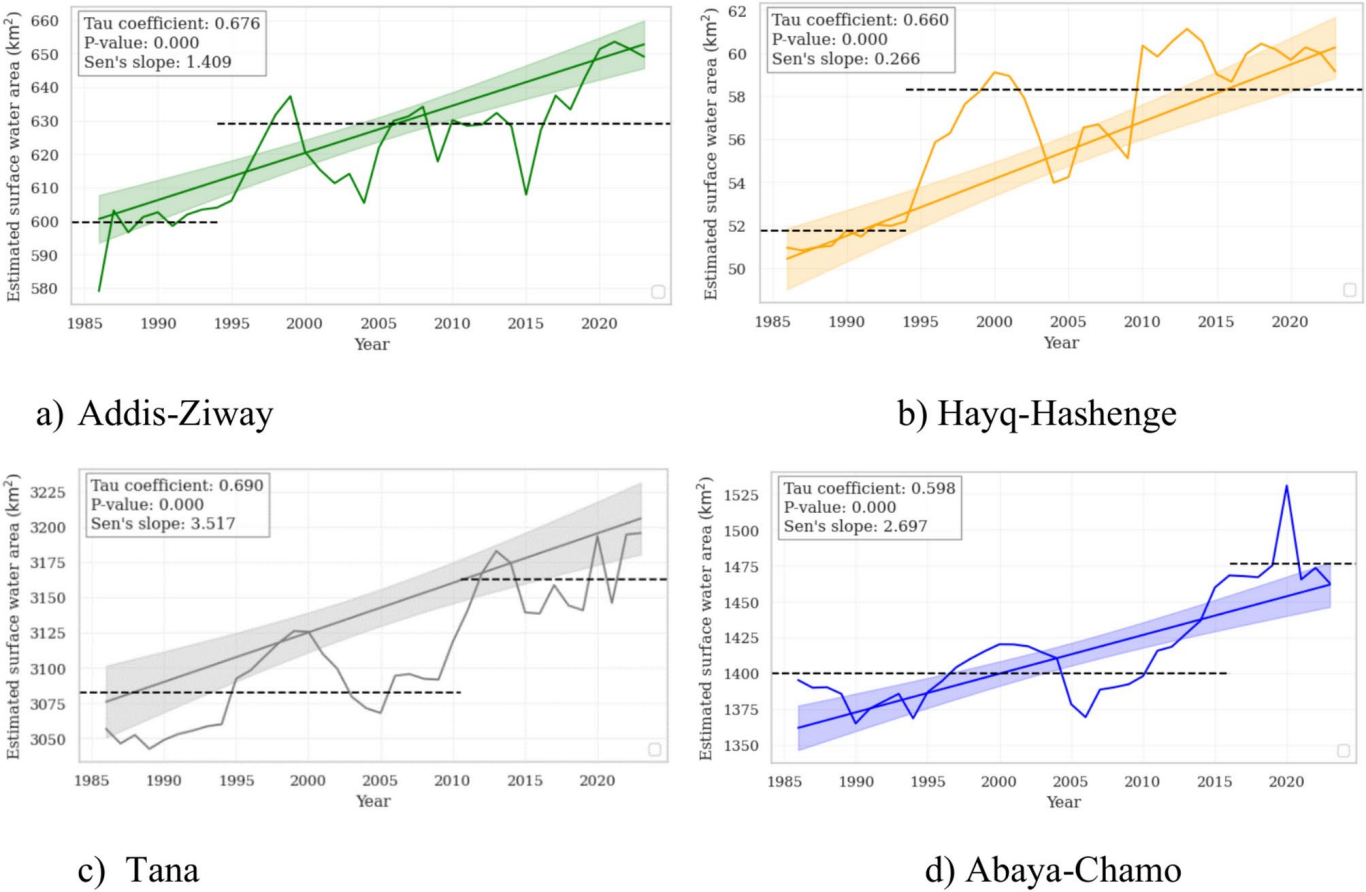
Analysis of long-term trends in surface water area. Tau fitted line with Kendall’s Tau coefficient, Sen’s slope, and p-values. The dotted black line shows the average surface water area before and after the year of change.

The long-term surface water dynamics at the four study sites show an increasing trend from 1986 to 2023, with notable inter-annual variability (Fig. 6). At Addis-Ziway, a significant increasing trend is observed with a Kendall Tau coefficient of 0.676 (p = 0.000) and a Sen’s slope of 1.409 km^2^ y^−1^. The average estimated surface water area increased by 5% after 2000 compared to before 2000 period. In the same way, the Hayq-Hashenge site shows a gradual increase with a rate of change of 0.266 km^2^ y^−1^. For both sites, a change in the trend is apparent at 1995. In Hayq-Hashenge the surface water area increases within 6 years by around 13% up to 58.3 km^2^. The average estimated surface water area shows an increase of about 2.6% after 2010 at the Tana site and a more significant increase of 5.4% after 2015 at Abaya-Chamo, highlighting a more pronounced expansion at the latter site. However, due to the size of the sites, changes before the turn of the millennium are not as pronounced as the changes from 2010 (Tana) and 2016 (Abaya-Chamo) onwards. Therefore, these results underscore significant spatio-temporal variations in surface water dynamics with general increasing trends.

### Surface water distribution and pattern

**Fig. 7:**
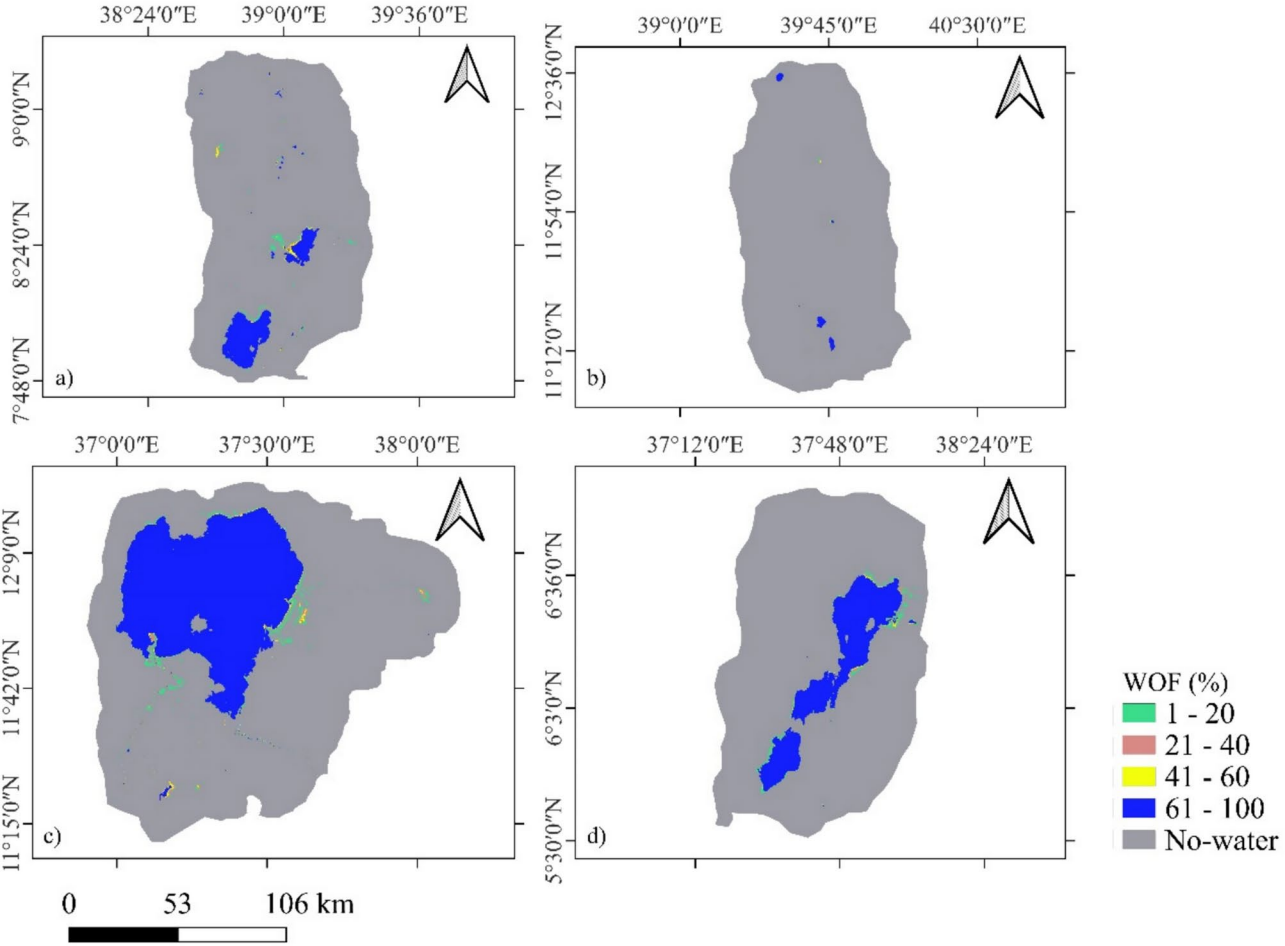
The Water Occurrence Frequency (WOF) from 1986 to 2023 in the four sites of (**a**) Addis-Zeway, (**b**) Hayk-Hashenge, (**c**) Tana and (**d**) Abaya-Chamo.

For the assessment of water occurrence over time we calculate the water occurrence frequency WOF and classify it into five classes of no water (0%) to full water coverage (100%) during the 38 years of observations. For the majority, a WOF 61–100% contributes to the majority of the surface water distribution across all sites (Fig. 7 and Table 1). The highest frequency is found for Tana with almost 94% followed by Abaya-Chamo with 91.6%. The two smaller sites have less stable surface area coverages with around 84% at WOF 61–100%. These sites show more dynamic pixels that often change between wet and dry conditions and which are less stable over time (WOF1-20% and 21–40%). As can be seen in Fig. 7, the WOFs show significant spatial variation across all sites. Lower WOF1-60% are spatially distributed and occur predominantly along the edges of areas with higher WOF61-100%, displaying a scattered spatial pattern. Overall, the high frequency of water occurrence across all sites represents the permanent water bodies, while the remaining proportions (1–60%) represent seasonal and temporary water bodies, including floodplains.

**Table 1:** Total (and percentage) surface water distribution by water occurrence frequency (WOF) classes from 1986 to 2023.

	Surface water area (km^2^) per WOF				
	Area [km^2^]	WOF1-20%	WOF21-40%	WOF41-60%	WOF61-100%
Hayk-Hashenge	63.8	5.0	3.1	2.1	53.6
Addis-Zeway	659.2	62.4	19.7	25.9	551.2
Abaya-Chamo	1525.0	83.4	23.7	21.6	1396.3
Tana	3218.4	125.7	46.9	30.6	3015.3

	Percent of surface water area (%) per WOF class				
	Area [km^2^]	WOF1-20%	WOF21-40%	WOF41-60%	WOF61-100%
Hayk-Hashenge	63.8	7.8	4.9	3.3	84.0
Addis-Zeway	659.2	9.5	3.0	3.9	83.6
Abaya-Chamo	1525.0	5.5	1.6	1.4	91.6
Tana	3218.4	3.9	1.5	1.0	93.7

## Discussion

### Machine learning for surface water monitoring

This study demonstrates the effectiveness of machine learning algorithms (GTB, RF, SVM) in surface water detection and monitoring. It is observed that all methods have a satisfactory classification accuracy of > 90% in terms of overall accuracy, producer’s accuracy and users’s accuracy. The robustness of the GTB is slightly higher, as evidenced by the overall accuracy rates of 94% to 98% across all sites. The effectiveness of GTB is probably due to its ensemble learning approach, which builds decision trees sequentially, each correcting the errors of the previous ones. This approach is particularly useful for detecting complex spectral patterns associated with water bodies. For example, Hastie et al., 2009 found that gradient boosting methods often outperformed RF in image classification. GTB achieved an average overall accuracy of 93.3% for mapping open and vegetated water bodies in the United States from 2017 to 2021^29^. However, we found that RF also has a commendable overall accuracy of over 90%. Similarly, an overall accuracy of 99% and user’s and producer’s accuracies of 96% and 87%, respectively, were observed for RF in the Murray-Darling Basin using Landsat data in Australia from 1986 to 2011^20^. Furthermore, high user’s (91.5 ± 2.5%) and producer’s (91.1 ± 6%) accuracies were achieved in the Cuvelai-Etosha Basin in Southern Africa from 1990 to 2021^43^. Thus, machine learning methods are effective for surface water detection, provide valuable spatio-temporal information for surface water area estimation, and offer excellent tools for long-term monitoring of different geographical locations in view of SDG 6.

### Comparison of surface water area estimates with the JRC dataset

Our estimated surface water area depicts a good agreement with the JRC dataset within the same aligned years, similar to other studies^31^. Despite this commonality and its good accuracy values, the JRC dataset has certain limitations at the local scale that make it less suitable to fully capture the surface water extent and interannual variability in our study areas from 1986 to 2023. This is due to the fact that the JRC method utilizes globally tuned models^43^. The GTB model, as confirmed by its high accuracy across all study sites, can effectively compensate for the data gaps in the JRC dataset. By providing a consistent and credible dataset, the model can support detailed analyses of interannual variability, trends, and drivers of surface water dynamics. This is crucial for understanding the implications of climate change, human activities, and natural variability on water resources. In conclusion, the GTB model effectively overcomes the limitations of the JRC dataset and provides robust and consistent spatiotemporal information for surface water monitoring across different geographic locations and time periods.

The strong agreement between our estimated surface water areas and the JRC dataset is evidenced by a high coefficient of determination (R^2^ > 0.9) and low RMSPE < 1% across all study sites. This underscores the reliability of the estimated surface water areas, and confirms the applicability of machine learning methods as was previously reported by) for the RF and the detection lake areas of Abiyata, Chamo, Ziway, Hawassa, and Abaya. Overall, we consider the JRC dataset to be a very useful reference for testing and developing alternative ways of estimating the spatio-temporal dynamics of surface water areas in data-poor regions. Despite the occasional missing values in the JRC dataset, the temporal patterns of our estimated surface water area and the JRC datasets remain consistent. This consistency suggests that our machine learning models can effectively compensate for data gaps in the JRC dataset and provide a consistent dataset of surface water monitoring for different geographical locations over time. Therefore, our approach is applicable and operational for different geographical regions and long-term monitoring of surface water in support of water resource management and conservation using publicly available data sources of GEE.

### Surface water dynamics and trends

The surface water dynamics of our study sites show a long-term increasing trend from 1986 to 2023, characterized by considerable inter-annual variability. The expansion of surface water at the Addis-Ziway site is influenced by anthropogenic factors, such as the Koka reservoir and associated dam infrastructure. In addition, the surface area of several lakes in the region, including Hora and Bishoftu, showed an increasing trend between 1984 and 2017^64^. Lake Ziway also showed a substantial long-term increase in surface area between 1985 and 2023^31^, although other studies have reported a consistent trend over earlier periods^65,66^. At the Hayq-Hashenge site, the surface water showed a gradual increase with a Sen’s slope of 0.266 km^2^ yr^-1^, with a notable shift in 1995 attributed to the construction of a dam near the town of Hara. However, the coverage of Lakes Hayq and Hardibo showed a declining trend^31,67^. For the Tana site, the average surface water area increased, with a notable increase after 2010. Prior studies have identified trends in the 1980s and early 1990s that show steady increases, preceded by significant variation about every five years^31^. At the Abaya-Chamo site, the statistically significant increasing trend was also observed by^68^, who reported surface water expansion between 2000 and 2022. In this study, we found that the construction of small and large dams and reservoirs across the study sites has contributed to an increase in surface water extent, along with the expansion of some natural lakes.

Dynamic changes in surface water extent is typical for many parts of the world. The permanent surface water extent in North America, for example, has increased by about 17,000 km^2^ from 1984 to 2015^1^, highlighting significant hydrological changes across the continent. Similarly, in Turkey, a long-term trend of increasing surface water extent has been observed due to the construction of new reservoirs and the development of surface water bodies^69^. Thus, dams and reservoirs have been identified as the main drivers of surface water increase worldwide^1^. In addition, surface water dynamics show considerable spatio-temporal variability in certain regions. For example, at the subcontinental scale in the semi-arid regions of Australia, high inter- and intra-annual variability in surface water extent has been observed between 1986 and 2011 ^20^. Similarly, annual sample-based estimates in the Cuvelai-Etosha Basin (CEB) of southern Africa show significant interannual fluctuations in surface water extent ranging from as low as 520.8 ± 375.7 km^2^ to as high as 12,372.3 ± 1,154.7 km^2^ between 1990 and 2021 ^43^. These spatio-temporal variability of surface water plays a key role in shaping water availability in many regions.

The results of the Mann–Kendall trend analysis do not show a general consistent temporal pattern in surface water dynamics across the four study sites. This indicates that local site characteristics, water management practices, and anthropogenic impacts play a significant role in determining surface water trends, potentially superimposing the broader impact of climate change. This underscores the importance in considering local factors influencing hydrological systems, especially in regions with diverse climatic and anthropogenic influences^70,71^. A one-size-fits-all method of water resource management may not be adequate to address the specific challenges of each study site. Instead, tailored site-specific water management strategies that take into account local hydrological, climatic, and socioeconomic conditions are needed to ensure sustainable water resource management^72^.

The predominance of high-frequency water occurrence across all study sites indicates the stability and persistence of surface water in these areas, reflecting the presence of permanent water bodies such as lakes, rivers, and reservoirs. These water bodies are maintained by consistent hydrological inputs, including upstream inflows and groundwater recharge^73,74^. Such areas are particularly crucial to the impacts of climate change and also vulnerable to human activities such as water withdrawals, land use changes, and dam construction, which can alter their spatial extent and hydrological regimes.

Surface waters with low to moderate water occurrence frequencies contribute less to the overall surface water distribution. These areas are characterized by the intermittent or seasonal presence of water, often associated with temporary water bodies, floodplains or wetlands. Such water bodies are highly dynamic and sensitive to both, climatic variability (e.g., droughts, floods) and anthropogenic activities. In particular, these areas are often affected by land use changes, such as the draining of wetlands for agriculture, which is particularly evident at the Hayq-Hashenge and Abaya-Chamo sites. Therefore, the surface water distributions and patterns associated with water frequency highlight the need for site-specific water management and conservation strategies that align with SDG6 monitoring efforts at the regional level.

## Conclusion

In this study, we provide and quantify spatio-temporal information for monitoring surface water extent using machine learning at four sites in Ethiopia from 1986 to 2023. GEE allows for the processing time series of surface water dynamics and monitoring using Landsat imagery with machine learning. It therefore has a great potential for monitoring surface water expansion also in remote areas. Our results confirm that machine learning using Landsat data produces reliable results for surface water monitoring and provides spatio-temporal information to support surface water management and water policy in Ethiopia.

The results of this study may have important implications for policy makers and water resource planners in Ethiopia, particularly in the context of sustainable water management and achieving SDG 6 at the regional level. The observed long-term increasing trend in surface water extent, together with high inter-annual variability, underscores the need for adaptive water management strategies. Policy makers should prioritize the development of Integrated Water Resource Management (IWRM) strategies, as local site characteristics, and anthropogenic influences affect surface water dynamics and trends. In addition, Ethiopia’s water policy should incorporate real-time surface water monitoring using machine learning-based remote sensing technologies and decision making to promote water conservation.

However, any approach to the dynamics of surface water extent that relies solely on remotely sensed imagery and historical data sets has difficulties in making fully-fledged future projections of surface water extent, as imagery and datasets are captured and collected from the past. Thus, for future projections, dynamic variables that change over time could complement the future spatio-temporal information on surface water extent. For example, hydro-meteorological variables from weather and climate projections or land use features from land use and land cover projections should enable such projections into the future. Therefore, future research needs to undertake large-scale assessments to provide a comprehensive national-level analysis of surface water dynamics and trends, taking into account the impacts of climate and land use change. This could be achieved by establishing an automated and operational system using GEE to provide near real-time spatio-temporal information on surface water extent to decision makers.

## Data Availability

The datasets generated during and/or analysed during the current study are available from the corresponding author on reasonable request.
